# Editorial: Traditional Medicine and Rheumatology

**DOI:** 10.3389/fphar.2021.707811

**Published:** 2021-06-24

**Authors:** Zhihua Yang, Xuan Tang, Huasheng Liang, Kaixin Gao, Maojie Wang, Xiaojuan He, Per-Johan Jakobsson, Runyue Huang

**Affiliations:** ^1^ Guangzhou University of Chinese Medicine, Guangzhou, China; ^2^ Guangdong-Hong Kong-Macau Joint Lab on Chinese Medicine and Immune Disease Research, Guangzhou University of Chinese Medicine, Guangzhou, China; ^3^ State Key Laboratory of Dampness Syndrome of Chinese Medicine, The Second Affiliated Hospital of Guangzhou University of Chinese Medicine, Guangzhou, China; ^4^ Guangdong Provincial Key Laboratory of Clinical Research on Traditional Chinese Medicine Syndrome, Guangzhou, China; ^5^ Center for Molecular Medicine, University Medical Center Utrecht, Utrecht, Netherlands; ^6^ China Academy of Chinese Medical Sciences, Beijing, China; ^7^ Karolinska Institutet (KI), Solna, Sweden

**Keywords:** disease-modifying antirheumatic drug (DMARD), traditional Chinese medicine, mechanism study, rheumatology, rheumatoid arthritis

Disease-modifying antirheumatic drugs (DMARDs) are often used in the treatment of several rheumatic diseases, such as Rheumatoid Arthritis (RA), Systematic Lupus Erythematosus (SLE) and Psoriasis. Methotrexate (MTX), the most common DMARD, even stands as the first-line therapy for RA (Smolen et al., 2020). However, due to the adverse events of synthetic DMARDs and high cost of biological DMARDs, the attainment rate of standardized therapies is not ideal. An increasing number of patients afflicted by rheumatic diseases are searching for help from Traditional Chinese Medicine (TCM). TCM has long been prescribed to prevent and treat rheumatic diseases with a good efficacy and little adverse reactions, but the mechanisms still remain largely ambiguous. On the one hand, we need robust evidence from high-quality clinical researches (including well-designed trials and meta-analysis). On the other hand, in-depth studies (such as multi-omics studies and network pharmacology) are needed to demonstrate the complex mechanisms of TCM. The aim of this editorial is to help understanding disease-modifying antirheumatic effects of TCM in rheumatic diseases.

Rheumatoid Arthritis (RA) is a systemic, autoimmune-related diseases, causing damage to bone and cartilage. Several studies have revealed the efficacy and safety of TCM in treating RA. It is found that most TCM compounds have good DMARD-likeness properties through literature screening (Li and Zhang, 2020). A meta-analysis (Daily et al., 2017) of randomized controlled trials on TCM compound GSZD (Guizhi-Shaoyao-Zhimu Decotion) showed that GZSD may have equal or superior effectiveness and safety when compared with conventional treatment. Clinical evidence from a monocenter, open-label, randomized controlled trial (Wu et al.) shows that HQT (a TCM formula) with MTX is a good therapeutic option in MTX-based treatment for RA. No statistical difference was observed between TCM formula-HQT with MTX group and Leflunomide with MTX group in terms of ACR20, ACR50, ACR70 and frequency of adverse events. The encouraging evidence has led to TCM treatments being increasing favored by clinicians.

However, the unspecific mechanism hinders the widespread and standardization of TCM. To explore the mechanisms of TCM treatment for RA, researchers (Wang et al.) performed a network pharmacology analysis and a Key gene Network Motif with Significant (KNMS) detection to figure out the most important components in three independent TCM formulas (Wang et al.). They found that three TCM formulas treats RA *via* VEGF signaling pathway, HIF-1 signaling pathway, PI3K-Akt signaling pathway, etc., which were further verified by *in vitro* cell experiments. The mechanisms of disease-modifying antirheumatic effects of TCM is also observed in animal experiments. For example, differentially expressed genes analysis, computational prediction and verification on collagen-induced arthritis (CIA) mice were utilized to reveal the mechanisms of TSPJ (a TCM formula) against RA. Led by a variety of bioinformatic cues, (Guo et al.) found that SRC and STAT3 may be the key targets of TSPJ through the VEGF and HIF-1 signaling pathways, thus suppressing inflammation and angiogenesis of RA. Moreover, (Du et al.) discovered that Tanshinone IIA, an isolated active ingredient in TCM, appears to act on MAPK, Akt/mTOR, HIF-1 pathways. In this study, Tanshinone IIA attenuated the inflammatory response (especially in terms of several inflammatory cytokines) in Avridine Induced Arthritis (AIA) mice and suppressed the activation of RA-FLSs induced by TNF-α. Above studies from the cellular level to the animal experiments convincingly demonstrate the antirheumatic effects of TCM in the treatment of RA. Most of them applied network pharmacology before verification. The work of Wang, et al. shed light on the antioxidant, and anti-inflammatory activities of TCM *via* examining the NO (nitric oxide) levels in different groups. Animal experiments by Guo et al. and Du et al. mimic the inflammatory status in RA patients. Compared to control intervention, TCM is not inferior in controlling inflammatory response and joint manifestations.

Bone destruction/erosion, an inevitable progress of RA, is a big challenge in RA treatments. (Cai et al.) addressed this challenge *via* a systematic review and meta-analysis, which provided literature evidence on bone-protecting efficiency of TCM in the treatment of RA. Bioactive compounds such as Triptolide and Celastrol extracted from TCM shows bone-protecting efficacy, and they have achieved good results from different aspects according to another meta-analysis study (Shi et al.).

Notably, the advantages of TCM treatment are also effective in other rheumatic diseases, such as Psoriatic Dermatitis and Osteoarthritis. Through flow cytometric analysis, CD4^+^ T cells and MDSCs (myeloid-derived suppressor cells) co-culture and a series of verification methods, (Deng et al.) revealed the complex mechanisms that TCM preparation PSORI-CM02 alleviated IMQ-induced psoriatic dermatitis and inhibited cell proliferation of Th17 by targeting M-MDSCs-induced (monocytic myeloid-derived suppressor cells) arginase-1.

The use of TCM in rheumatic diseases dates back to thousands of years ago. Compared with standardized treatment, TCM has the advantages of low cost, low side effects, and flexible medication. Multi-component, multi-target and multi-pathway allows TCM to modify rheumatic diseases in different ways, which also makes it difficult to assess efficacy and figure out the specific mechanisms. Current studies on TCM in rheumatic diseases varied in study design and research findings. Most of them merely focused on the relationship “drug-gene-protein-disease,” but they seldomly illustrate the specific pharmacological actions. Weak foundation in prior work results in this situation. TCM researches is dwarfed by conventional DMARDs researches in pharmacology, toxicity and pharmacokinetics. Since there were plenty of literature studies, we need to form hypotheses or generate specific research directions according to former findings.

With the help of new and high-tech methods, current mechanism researches of TCM against rheumatic diseases has been developing. We believed that current findings are only a tip of the iceberg, but the unknown mechanisms and unspecific efficacy holds a great potential in Rheumatology.
